# A Student-Led Campaign to Help Tackle Neglected Tropical Diseases

**DOI:** 10.1371/journal.pmed.0040241

**Published:** 2007-07-24

**Authors:** Sandeep P Kishore, Prabhjot S Dhadialla

## Abstract

The authors propose that innovative student-led campaigns to address neglected diseases can and do make a practical difference.

The neglected tropical diseases (NTDs) are a group of chronic infections that are often considered together because they primarily affect one billion of the world's poorest people and attract little attention from the global medical community [1]. The United Nations Millennium Development Goals (http://www.un.org/millenniumgoals/) call for increased funding for HIV/AIDS, malaria, and “other diseases” (including the NTDs), but this call has yet to yield significant support for NTD control [2].

**Box 1. Student-Driven Opportunities to Address NTDs**

After the forum, students created opportunities with local collaborators for experiences in sub-Saharan Africa. One such partnership was with the Millennium Cities Initiative (http://www.cpii.columbia.edu/projects/; see Figure 1) and the Earth Institute of Columbia (http://www.earth.columbia.edu/).

The Millennium Cities Initiative advises seven mid-sized cities across sub-Saharan Africa on how to achieve the Millennium Development Goals, providing research and policy analysis. Tri-I medical students will assist this initiative by developing and carrying out needs assessments in areas of public health (including goals such as reducing child mortality by two-thirds and maternal mortality by three-quarters, and reversing the spread of HIV/AIDS and malaria). With the information gleaned during these assessments, students will publish a report with the United Nations and/or the Earth Institute on their work. It is hoped that these reports will help policy makers in each African Millennium City to decide for themselves what to focus on in the short, medium, and long term. The reports may also help national governments and the donor community to estimate the resources that will be required for these cities to reach the Millennium Development Goals.

Universities are uniquely positioned to provide biomedical and clinical expertise, and they boast core missions that seek to promote the public welfare. The university motto of the Rockefeller University, for instance, is: *“pro bono humani generis,”* or “science for the benefit of humanity” (http://www.rockefeller.edu/). In this article, we propose that innovative student-led campaigns to address NTDs can and do make a practical difference. We discuss these efforts at our universities.

## Our Student-Led Movement Generates Momentum

In coming to medical school, several of us had like-minded interests in global health and were committed to making a practical difference. We formed a small caucus of medical and graduate students at the Weill Cornell Medical College, Rockefeller University, and Sloan-Kettering Cancer Institute Tri-Institutional (Tri-I) campus in New York to explore the issue. We partnered with a growing student-led movement across universities, called Universities Allied for Essential Medicines [3]. This movement recently catalyzed the creation of the Philadelphia Consensus Statement (http://www.essentialmedicine.org/cs/) to promote equal access to university discoveries in the developing world and to promote university research on global health concerns. We brought the movement to our local campuses to find how our homegrown resources could be best leveraged.

As student interest was mounting, we saw an opportunity to engage the biomedical community when one of our universities (Cornell University) issued a letter in November 2006 calling for innovative proposals to help promote development efforts in sub-Saharan Africa. Since much of the burden of the NTDs is in Africa, we used this call for proposals to organize student, faculty, and institutional support.

We planned a comprehensive one-day forum on neglected diseases to open a local dialogue on the issue. We weren't expecting very much interest, and so we were stunned by the community response. Nearly overnight, we had found that our student-led forum itself had caught the eye of the leading Tri-I research faculty, clinicians, philanthropists, and locally based collaborators, such as Médecins Sans Frontières, the Earth Institute at Columbia University, and the New York Academy of Sciences. Furthermore, our forum provided the nucleus of a community for students and faculty with similar interests.

The forum placed NTDs squarely in the limelight and asked how universities could help to tackle these diseases of the developing world. A distinguished panel of speakers provided updates and commentaries on their work, including Peter Hotez of the Global Network for Neglected Tropical Disease Control (http://gnntdc.sabin.org/) and Editor-in-Chief of *PLoS Neglected Tropical Diseases* (http://www.plosntds.org/), Nobel laureate and Public Library of Science co-founder Harold Varmus, Ellis Rubinstein, the President of the New York Academy of Sciences, Joanna Rubinstein, Chief of Staff at the Earth Institute, and global health pioneers Carl Nathan and Warren Johnson. Video podcasts of their talks are freely available at http://collections.plos.org/plosntds/uaem.php. The Cornell administration and dean took a special interest in our movement, quickly grasping the essence of our call to arms. Now the question became: how exactly can universities help?

## Research

As highlighted in Peter Hotez's keynote address, it is clear that universities are uniquely positioned to promote scientific research on the neglected diseases of poverty. Unfortunately, among the over 1,200 chemical entities commercialized from 1975 to 1997, only 13 (1%) were specifically for tropical diseases [4]. At Tri-I, for instance, there are less than five basic scientists who work on an NTD, compared with dozens of researchers working on other infectious diseases such as HIV/AIDS. Universities could therefore play an important role in tackling NTDs by expanding their research base to develop a new set of control tools for NTDs. For example, universities could earmark monies derived from capital campaigns to support specific global health or NTD research initiatives. Of the over 50 universities with endowments of at least $1 billion (http://chronicle.com/indepth/endowments/), only a handful have such initiatives.

Students and members of the Tri-I community support Harold Varmus' suggestion to implement one-year exchange fellowships between the United States and foreign scientists. This program, the Global Science Corps (http://www.globalsciencecorps.org/) and others like it are tied to pre-existing institutional infrastructures [5]. Such “twinning” with international research partners provides unique exposure to different systems of science for American and overseas students.

## Clinical Training

Weill Cornell already provides support for the 40% of its medical students who wish to spend part of their clinical electives abroad. Reciprocally, clinical exchange programs exist at all stages of training, such as an exchange between African and South American students, residents, and fellows in the medicine department. As a result of partnerships forged at the Tri-I forum, we are adding to these exchange programs by developing an innovative health-based partnership with the Millennium Cities Initiative (Box 1), where students will work with local public health experts to complete needs assessment surveys of the cities.

Our forum highlighted international opportunities that may not have had strong public exposure. For instance, a 45-year-Old collaboration between Weill Cornell and the Federal University of Bahia in Salvador, Brazil, focusing on the NTD leishmaniasis, has yielded over 250 peer-reviewed publications and a $32 million Gates Foundation grant for a leishmaniasis vaccine. And a long-standing clinical site in Port-au-Prince, Haiti that traditionally focuses on HIV/AIDS presents an excellent opportunity for students to also address NTD control efforts. In these partnerships, clinical and research studies and services are jointly designed and executed by all participants.

**Figure 1:**
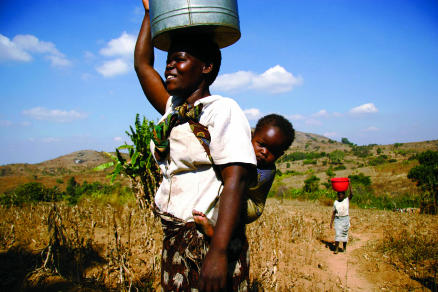
The Millennium Villages Project Photo: Yoshi Shimizu/International Federation of Red Cross and Red Crescent Societies

With the support of philanthropic partners such as the TOUCH Foundation (http://www.touchfoundation.org/), Weill Cornell has co-developed a medical school program in Mwanza, Tanzania with an existing medical institution, the Bugando University Medical Center. This year, the first Cornell students will arrive in Tanzania to spend clinical and research time on NTDs, mentored by Cornell physicians. This process will be reciprocally strengthened by existing NTD expertise at the Bugando University Medical Center. By highlighting these opportunities for NTD-specific experiences abroad, students will be able to more effectively target their interests in this direction, and (more importantly) to establish mutually productive, sustainable partnerships with health workers abroad.

## Education

We have developed an academic-themed community on NTDs for Tri-I. Within this structure, we have already implemented a journal club that assesses the science related to NTDs and developed a clinical seminar for medical students that addresses gaps in the medical curriculum related to global health and NTDs (Box 2). Our journal club will begin annotating studies on NTDs published in *PLoS ONE* (http://www.plosone.org/), and will also annotate papers in *PLoS Neglected Tropical Diseases* once it has launched.

To sustain students' interest we have developed a seminar series on NTDs, which has featured a diverse array of speakers, such as Jeffrey Sachs, Special Advisor to the United Nations Secretary-General, and Rashmi Barbhaiya, chief executive officer of Advinus, an Indian-based pharmaceutical firm interested in NTDs. Peter Hotez has commented that given all the available resources and expertise at our institutions, we could go a step further towards developing a formal educational program—a new biomedically based, interdisciplinary masters degree in global health or parasitology, for example.

**Box 2. Student-Created NTD Short Course: Global Challenges of Managing Neglected Diseases**

In order to inform students about the clinical and global health dimensions of managing NTDs, we have designed a series of six lectures targeted at medical students in their pre-clinical years. Traditional medical curricula de-emphasize NTDs, and curricula that do cover NTDs rarely address non-clinical and research aspects. Our course will not only cover clinical pathogenesis of selected NTDs, but aims to contextualize the challenges of managing these diseases in a complex political, economic, and logistical landscape. We have drawn upon broad expertise and personal experiences to introduce these NTDs in the context of real case studies. The lecture series will actively engage pre-clinical students by asking all participants to collectively produce a document that explores ways our universities can apply their resources to provide concrete solutions to tractable problems related to specific NTDs.

## Conclusion

University students are by no means passive players in the efforts to increase biomedical attention to the developing world. In the case of NTDs, our efforts are now catalyzing commitment from all university levels. In the long term, we aim to develop a Tri-I Initiative for Diseases of the Developing World, with increased commitment at the bench, bedside, and all stages in between. We now look forward to developing our movement with others across the globe.

## Abbreviations

NTD: neglected tropical disease
Tri-I: Tri-Institutional

## References

[pmed-0040241-b001] Molyneux DH, Hotez PJ, Fenwick A (2005). “Rapid-impact interventions”: How a policy of integrated control for Africa's neglected tropical diseases could benefit the poor. PLoS Med.

[pmed-0040241-b002] Sachs J, Hotez PJ (2006). Fighting tropical diseases. Science.

[pmed-0040241-b003] Chokshi DA (2006). Improving access to medicines in poor countries: The role of universities. PLoS Med.

[pmed-0040241-b004] Pecoul B, Chirac P, Trouiller P, Pinel J (1999). Access to essential drugs in poor countries: A lost battle?. JAMA.

[pmed-0040241-b005] Varmus H (2002). Building a global culture of science. Lancet.

